# Research on risk factors of human stampede in mass gathering activities considering organizational patterns

**DOI:** 10.3389/fpubh.2025.1555735

**Published:** 2025-04-08

**Authors:** Ying Lu, Jingwen Wang, Weihui Dai, Bingxue Zou, Xiaofang Lu, Weisheng Hong

**Affiliations:** ^1^School of Resources and Environmental Engineering, Wuhan University of Science and Technology, Wuhan, China; ^2^Safety and Emergency Research Institute, Wuhan University of Science and Technology, Wuhan, China; ^3^School of Management, Fudan University, Shanghai, China

**Keywords:** mass gathering, organizational pattern, spontaneous gathering, risk factors, unsupervised clustering algorithms

## Abstract

**Introduction:**

Mass gathering activities are often accompanied by safety risks especially overcrowding, since the characteristics of risk factors may differ under various organizational patterns. However, this issue has not been sufficiently studied, which could lead to cognitive biases in understanding the risks associated with various mass gathering activities, thereby affecting the effectiveness of preventive measures.

**Methods:**

This study investigates the risk factors of human stampede in mass gathering activities, with a particular focus on analyzing the influence of different organizational patterns on these risks and their implications for safety management. By combining Grounded Theory and Iterative Self-Organizing Clustering Algorithm (ISODATA), 209 overcrowding cases were coded to create a dataset containing risk factors.

**Result:**

The organization coefficient was proposed to characterize the degree of risk in different clusters, and the clustering results revealed three types of organizational patterns including organized( $O_{31}$ =1.9174), applied( $O_{82}$ =2.9831), and spontaneous( $O_{73}$ =4.4327).

**Discussion:**

The results indicate significant differences between the three organizational patterns in terms of aggregation causes, layout characteristics, and risk levels, which directly impacts the safety of mass gathering activities. Furthermore, similarities are observed in terms of triggering behaviors, knowledge, awareness, and management across the four categories. This study provides theoretical evidence for risk prevention and safety management of human stampede in mass gathering.

## 1 Introduction

With the recovery of the tourism industry and the diversification of travel destinations, the mobility of tourists has been continuously increasing (1). Popular scenic spots are drawing an increasing number of tourists, especially during holidays and peak seasons, when the influx of tourists becomes particularly obvious (2). Nevertheless, the surge in visitor numbers has also revealed a series of issues, among which the most prominent is overcrowding (3). Against this backdrop, the phenomenon of spontaneous crowd gatherings has been on the rise, particularly evident at large-scale music festivals (1, 4), religious events (5), and street activities (6). While these unplanned activities offer opportunities for free communication and experiences, they also pose safety risks. For instance, on October 29, 2022, a human stampede disaster occurred in Seoul, South Korea, originated from thousands of people crowding into the Itaewon district for the Halloween festivities. The disaster resulted in a total of 158 fatalities and 196 injuries (6), which is a typical overcrowding phenomenon that occurred in a spontaneous activity. Due to the undetermined number of participants, uncertain timing, and unspecified form of gathering, the characteristics of risk factors during spontaneous activities differ from those in traditionally organized activities (7). Therefore, it is of great importance to study the risk characteristics in mass gathering under different organizational patterns.

To examine the influence of organizational patterns on mass gathering activities, it is essential to categorize activities based on their organizational patterns. Previous studies on activities primarily focused on planned rather than unplanned activities, often targeting specific settings or places such as stadiums (8), temples (9, 10), subways (11, 12), schools (13, 14), etc. But in fact, large assemblies can occur spontaneously, or can be organized for specific purposes. By comprehensively considering the possible organizational patterns that various activities may present (15), mass gathering activities are categorized into three types which include the type of organized, applied, and spontaneous. Among them, organized activities are generally hosted by the government, with clear organizers and risk prevention measures (16). These activities often involve structured itineraries, regulated crowd behavior, and better-managed safety infrastructure, which help mitigate risks (13). In these environments, crowd behavior is more standardized and safety measures are more systematically implemented. Applied activities are organized by social organizations, individuals, or scenic spots with government approval and also have organizers. Spontaneous activities are gatherings of people, occurring simultaneously at the same time and in the same place for a specific purpose, with no organizers and relatively weak risk reduction measures (17).

Upon categorizing organizational patterns, it is crucial to understand the factors contributing to the overcrowding and analyze how different organizational patterns impact the risk characteristics of these activities (18). The selection of comprehensive risk factors and the choice of appropriate analytical methods are two important considerations for establishing an accurate risk model to study the mechanism of overcrowding.

As for risk factors, scholars have used various methods such as proportional statistical analysis (11, 12, 19, 20) and Accident Causation Theory (21–24) to analyze these factors through case studies. For instance, Illiyas et al. (20) identified risk factors like crowd density, collapse of temporary structures, rumors, and accidental notifications in 34 religious festival stampede accidents in India. Helbing and Mukerji (23) analyzed the 2010 Germany music festival stampede, attributing it to panic-induced crowding and a domino effect of panic spread. Although methods like HFACS (24, 25), Management Oversight Risk Tree (MORT) (26, 27), and Accident Causation Sequence Theory (28, 29) are increasingly used in research on overcrowding, these frameworks are predefined and often do not consider organizational patterns. Grounded Theory, which uses inductive reasoning based on existing cases (30), is well-suited for exploring new areas of research (31, 32). For example, Xu et al. (33) constructed a conceptual model of risk factors based on 59 air traffic controller accidents. Li et al. (34) used Grounded Theory to examine 135 industrial accidents, uncovering novel structural factors in industrial accidents. Therefore, the Grounded Theory is highly suitable for exploring situations where risk factors are unknown in the context of overcrowding.

In regard to analytical methods, while Grounded Theory proves instrumental in comprehensively exploring risk factors, its limitation lies in its qualitative nature, lacking the ability to quantitatively measure inter-factor correlations. Currently, multi-factor network research methods such as Complex Network Model (35–37), Interpretive Structural Modeling (ISM) (38), Bayesian Network (8, 39, 40) etc. have been used to investigate the correlation among multiple factors in overcrowding. For example, Siyu et al. (41) who utilized ISM analysis, delineated the hierarchical relationships among risk factors. Nonetheless, challenges arise due to subjectivity in assessing the connections between influencing factors. Lu et al. (40) drawing from 46 accident cases, devised a risk analysis model for stadium overcrowding, by leveraging Bayesian networks and triangular fuzzy numbers. They identified 24 risk factors, categorizing them into distinct states. Results indicate that factors such as audience emotions, vulnerable groups, and counterflow crowds are more prone to causing overcrowding incidents. While network modeling research facilitates the quantitative analysis of inter-factor correlations and their impact on the risks of overcrowding, it is predominantly within homogeneous contexts, limiting its capacity for differentiated research on risk factors across diverse organizational patterns.

In fact, addressing the heterogeneity of risk factors for different types of mass gathering stands out as a pivotal concern (42, 43). Some researchers segment cases based on expert experience, but cannot guarantee the grouping of samples from the same type of activities (44). Another portion of studies utilizes specific analytical techniques such as clustering algorithms (45), random parameter fractional logit models (46–48), graph convolutional neural networks GCN algorithm (49), random forests (RF) (50), deep neural networks (DNN) (51), as auxiliary tools for case categorization. Compared with pre-defined classification rules or specific model methods, clustering algorithm can aggregate based on the similarity and difference of data itself, maximize the homogeneity within groups and heterogeneity between groups, reveal potential and undiscovered characteristics or laws, and help to analyze and explain the accident classification problem more comprehensively (52, 53). Therefore, on the base of understanding the risk factors, applying clustering algorithms to analyze the characteristics of risk factors under different organizational patterns in mass gathering activities is applicable.

The Iterative Self-organization Algorithm (ISODATA) is based on the k-means algorithm, with additional operations for “merging” and “splitting” clusters. It also incorporates control parameters to manage the algorithm’s execution, enabling it to handle the clustering needs of high-dimensional, large-scale datasets (52, 54). This algorithm is particularly well-suited for situations with numerous risk factors, high dimensionality, and complexity, such as in mass gathering, as it can effectively identify and manage patterns and structures in high-dimensional data. A key focus is how to integrate grounded theory with ISODATA to establish a risk model. Lu et al. (53) proposed a method that combines iterative self-organizing data analysis (ISODATA) with fuzzy theory to explore the similarities and differences in risk factors for overcrowding cases within different risk level clusters. ISODATA analysis facilitates the effective identification of characteristic information associated with heterogeneity between clusters. However, how to distinguish clusters from the view of organization pattens and determine the heterogeneity of risk characteristics under different mass gathering activities is an unresolved issue.

To address the aforementioned issues, this study introduces a method that integrates Grounded Theory and ISODATA to analyze the characteristics of risk factors for overcrowding under different organizational patterns. The method collects the textual data of 209 overcrowding cases occurring from 2000 to 2020, extracting information to establish a comprehensive dataset delineating the risk factors contributing to overcrowding occurrences. Clustering models and organization coefficients are used to classify three distinct types of mass gathering. Furthermore, the characteristics of risk factor in mass gathering across the three organizational pattens are quantitatively compared and discussed. This research reveals the causative mechanism of overcrowding, contributing to the enhancement of risk prevention measures for sustainable travel.

## 2 Methodology

### 2.1 Sample information collection

The keywords “stampede” or “crowded” and “mass gathering” or “overcrowding” are employed for searches across search engines including Google, Bing, or Baidu. A total of 383 investigative reports and related news reports on overcrowding cases worldwide in recent years are collected. The data pertaining to overcrowding cases is authentic, comprehensive, and reflective of current social development and crowd activities. Ultimately, 209 overcrowding cases occurring between 2000 and 2022 are chosen as the samples for this research.

### 2.2 Identification of risk factors in mass gathering

Grounded Theory refers to the analysis and organization of primary data to establish a theoretical system or obtain conclusions (55). The whole process involves hierarchically coding the collected data and information. First, the open coding phase entails organizing the collected materials into text format and importing them into the software NVivo 11 to create free nodes for words and phrases related to overcrowding cases. Totally, 3,186 reference points are established, and similar concept points were repeatedly compared and combined to form 79 concepts. Secondly, all free nodes were integrated to complete the encoding, and nodes with the same or similar meanings are attributed to a tree node, thus obtaining axial coding. Then, all the tree nodes were further analyzed and generalized to obtain new selection codes and form the structured coding results. Focusing on the logical relationship between preliminary categories, the initial concepts are comprehensively summarized and classified, and finally 15 core categories and 57 spindle codes are extracted. An excerpt of the coding process is shown in Table 1. At the end, a coding saturation test is conducted, in which the findings are determined to be saturated by coding new accident cases on a level-by-level basis, if no new conceptual descriptions or categorized concepts appear in the data (56).

**Table 1:** Scope formation and implementation coding process (excerpt).

Press coverage of human stampede accident (typical description)	Scoping (open coding)	Spindle type coding	Selective coding
Paralysis on the part of the Government and the police, who failed to coordinate carefully with organizers on safety and security measures, and failure to maintain good security and order in the organization of events	Lack of careful coordination of security measures, failure to maintain good security order	Weak on-site crowd management capabilities	Security management aspects
No effective security system in the stadium	No effective security system	Inadequate emergency response system in crowded places	
More than 50 police officers tried their best to divert the situation, but failed to do so, eventually causing a stampede of casualties	Evacuation did not work	Poor crowd dispersal at the scene	
In order to prevent stampede, the security guards hired on an *ad hoc* basis at the amusement park signaled to the crowd to sit down and wait in front of them, a gesture that was misinterpreted as the “start of the procession.” The thousands of Hajj pilgrims behind them, unaware of what was going on in front of them, continued to move forward.	Misinformation transfer, failure of information transfer within a crowd	Crowd information exchange failures (within crowd, between crowd and managers)	
The school arranged only one on-site caretaker to carry out security patrols and on-site management, making it difficult to monitor all the students descending the stairs. There was a lack of specialized personnel to maintain order, while as many as 5,000 people participated in the Open-Door Day activities, there were only about 200 police officers to maintain security	Lack of specialized personnel to maintain order, insufficient police presence to maintain security	Inadequate security force	

### 2.3 Structure of feature vectors for human stampede in mass gathering

The core principle underlying cluster analysis involves the comparison of distinct attributes among categorized entities, with the accuracy of clustering outcomes significantly reliant on the eigenvectors. Consequently, the selection of reasonable and representative quantitative indicators stands as a crucial prerequisite for effective clustering. According to the coding results of Grounded Theory analysis, combined with the literature addressing risk factors in human stampede accident (9, 10, 19, 20, 23, 40, 53, 57–59), comprehensive characterization variables are identified to encapsulate facets of accident data, encompassing temporal, spatial, risk factors and organizational patterns. Building upon this, an overcrowding cases dataset was constructed by quantizing the eigenvectors (refer to Table 2), with detailed information available in Appendix A.

**Table 2:** Risk factors of human stampede accident eigenvectors and grading.

Site	Aggregation cause	Organizational pattern	Layout character	Characteristics of open place	Time	Risk grade	Risk factors of direct causes				Risk factors of indirect causes			Risk factors of root cause
							Unsafe behavior			Unsafe object	Knowledge	Consciousness	Psychology	Management
							Triggering behavior	Human structure	Crowd flow					
School education (1) S1	Religious activities (1) A1	Organized (1) O1	Narrowing (1) L1	Indoor (1) V1	Morning (1)T1	R1 (0) (1)	Quarrels, fights, terrorist attacks (1) B1	Mainly elder-age (1) H1	Hetero-hedging of the flow of people (1) F1	Unreasonable number and width of safety exits (1) E1	Lack of knowledge of accident hazards (1) K1	Weak security awareness (1) C1	Panic (1) P1	Weak crowd management capacity (1) M1
Cultural Communication (2) S2	Sports competitions (2) A2	Applied (2) O2	Ramps (2) L2	Outside (2) V2	Afternoon (2) T2	R2 (1 ~ 10) (2)	Falling, squatting (2) B2	Mainly middle-aged (2) H2	Velocity differences in the isotropic population (2) F2	Steps with large height differences and narrow widths(2) E2	Weak accident response skills (2) K2	Poor emergency preparedness (2) C2	Conformity psychology (2) P2	Inadequate emergency response system in crowded places (2) M2
Traffic hub (3) S3	Celebrations (3) A3	Spontaneous (3) O3	Alleyway (3) L3		Evening (3) T3	R3 (11 ~ 100) (3)	Mischievous (3) B3	Mainly teenagers (3) H3	Localized density spikes (3) F3	Slippery roads (3) E3	Not relevant (3) K3	Not relevant (3) C3	Competitive psychology (3) P3	Poor crowd dispersal at the scene (3) M3
Tourist attraction (4) S4	Holidays (4) A4		Bridge (4) L4		Unknown (4) T4	R4 (101 ~ 1,000) (4)	Violent law-enforcement (4) B4	Unknown (4) H4	Not relevant (4) F4	Road blockage, unreasonable gradient, width (4) E4			Feverish psychology (4) P4	Crowd information exchange failures (4) M4
Hotel and Dining (5) S5	Concerts (5) A5		Stairs (5) L5			R5 (over 1,000) (5)	Rumors (5) B5			Failure of safety precautions (5) E5			Not relevant (5) P5	Security is inadequate (5) M5
Entertainment and Leisure (6) S6	Charity and Promotions (6) A6		Entrance/exit (6) L6				Not relevant (6) B6			Poor lighting (6) E6				Not relevant (6) M6
Urban public spaces (7) S7	Education and training (7) A7		Not relevant (7) L7							Sudden occurrence of a disaster (7) E7				
Mall Promotion Place (8) S8	Sudden Disaster (8) A8									Overloading of premises capacity (8) E8				
										Not relevant (9) E9				

### 2.4 Organizational patterns clustering of human stampedes in mass gathering activities

#### 2.4.1 Establish the clustering model for human stampede based on ISODATA

ISODATA is an improved clustering algorithm. Compared with traditional weighted clustering algorithms, which rely heavily on initial values (60), it adds merging and splitting operations on the basis of the traditional weighted clustering algorithm (54). The calculation process can automatically merge and segment clusters, avoiding the impact of unreasonable selection of initial clustering centers on the results. The algorithm rules are clear and easy to implement by computer (61). Therefore, in order to determine the optimal clustering center, the ISODATA clustering algorithm was chosen to cluster the dataset, and the optimal clustering number was selected in comparison.

Step of using ISDODTA to establish the clustering model of human stampede in mass gathering activities.

##### 2.4.1.1 Step 1: initial cluster division

1. Input 209 pattern samples $\left\{x_{i},i=1,2,\ldots ,N\right\}$ ; preselect $N_{c}$ initial cluster centers $\left\{z_{1},z_{2},\ldots z_{N_{c}}\right\}$ , which may not be equal to the required cluster centers. The initial cluster centers can be arbitrarily selected from the sample. The relevant preselected values are listed in Table 3.
2. Assign N pattern samples to the nearest cluster $S_{j}$ , if $D_{j}=\min\left\{x-z_{i},,,i=1,,,2,,,\ldots N_{c}\right\}$ , that is the distance of $x-z_{i}$ is the smallest. Then $x\epsilon S_{j}$ .
3. If the number of samples in $S_{j}$ is $S_{j}<\theta_{N}$ , then the sample subset is cancelled, and $N_{c}$ is reduced by 1.

**Table 3:** Threshold parameters.

Symbol	Meaning
K	Number of clusters
$\theta_{N}$	Minimum number of data in a cluster
$\theta_{S}$	Standard deviation based on distance
$\theta_{c}$	Minimum distance between each two clusters
L	Largest number of clusters to merge or to slit every time
I	The largest iteration times

##### 2.4.1.2 Step 2: cluster refinement and distance calculation

1. Revise each cluster center the calculation expression of *Z_j_* is shown in Equation 1

(1) $$z_{j}=\frac{1}{N_{j}}\sum_{x\in S_{j}}x,j=1,2,\ldots N_{c}$$

2. Calculate the average distance between each clustering domain $S_{j}$ pattern sample and each cluster center Refer to Equation 2 for calculation.

(2) $$\bar{D_{j}}=\frac{1}{N_{j}}\sum_{x\in S_{j}}||x-z_{j}||,j=1,2,\ldots ,N_{c}$$

3. Calculate the total average distance between all model samples and their corresponding cluster centers. The specific computational formula adopted here is Equation 3

(3) $$\bar{D}=\frac{1}{N}\sum_{j=1}^{N}N_{j}\bar{D_{j}}$$

##### 2.4.1.3 Step 3: discriminate split, merge and iterative operations

1. If the number of iteration operations has reached 1, that is, the last iteration, set $\theta_{c}=0$ , and go to the step 5;
2. If $N_{c}\leq \frac{K}{2}$ , that is, the number of cluster centers is less than or equal to half of the specified value, go to step 4 to split the existing clusters;
3. If the number of iterative operations is an even number, or $N_{c}\geq 2K$ , do not split and go to the fifth step; otherwise (it is neither an even number of iterations nor satisfy $N_{c}\geq 2K$ ), go to the fourth step to split deal with.

##### 2.4.1.4 Step 4: assessing the dispersion of cluster samples

1. Calculate the standard deviation vector $\sigma_{j}={\left(\sigma_{1j},\sigma_{2j},\ldots ,\sigma_{nj}\right)}^{T}$ of the distance of each cluster sample, where the components of the vector are

(4) $$\sigma_{ij}=\sqrt{\frac{1}{N_{j}}\sum_{k=1}^{N_{j}}{\left(x_{ij}-z_{ij}\right)}^{2}}$$

In Equation 4, i=1,2,…,n is the dimension of the sample feature vector, $j=1,2,\ldots ,N_{c}$ is the number of clusters, and $N_{j}$ is the number of samples in $S_{j}$ .

2. Find the maximum component in each standard deviation vector $\left\{\sigma_{j},j=1,2,\ldots ,N_{c}\right\}$ , represented by $\left\{\sigma_{\mathrm{jmax}},j=1,2,\ldots ,N_{c\text{.}}\right\}$
3. In any maximum component set $\left\{\sigma_{\mathrm{jmax}},j=1,2,\ldots ,N_{c}\right\}$ , if there is $\sigma_{\mathrm{jmax}}>\theta_{S}$ , and one of the following two conditions is met at the same time: $\bar{D_{j}}>\bar{D}$ and $N_{j}>2\left(\theta_{N}+1\right)$ , that is, the total number of samples in $S_{j}$ exceeds the specified value More than doubled; $N_{c}\leq \frac{K}{2}$ splits $z_{j}$ into two new cluster centers and they are $z_{j}^{+}$ and $z_{j}^{-}$ , and $N_{c}$ is increased by 1. The component corresponding to $\sigma_{\mathrm{jmax}}$ in $z_{j}^{+}$ is added to $k\sigma_{\mathrm{jmax}}$ , and the component corresponding to $\sigma_{\mathrm{jmax}}$ in $z_{j}^{-}$ is subtracted from $\sigma_{\mathrm{jmax}}$ . If the split operation is completed in this step, go to 2) of the first step, otherwise continue.

##### 2.4.1.5 Step 5: cluster center distance calculation and merging

1. Calculate the distance of all cluster centers using Equation 5

(5) $$D_{ij}=||z_{i}-z_{j}||,i=1,2,\ldots N_{c}-1,j=i+1,\ldots ,N_{c}$$

2. Compare the values of $D_{ij}$ and $\theta_{c}$ , and arrange the values of $D_{ij}<\theta_{c}$ in ascending order of the smallest distance, namely $\left\{D_{i1j1},D_{i2j2},\ldots D_{\mathrm{iLjL}}\right\}$ .

Where $D_{i1j1}<D_{i2j2}<\ldots <D_{\mathrm{iLjL}}$

3. Combine the two cluster centers $Z_{ik}$ and $Z_{jk}$ with a distance of $D_{\mathrm{ikjk}}$ , and get the new center as:

(6) $$z_{k}^{*}=\frac{1}{N_{ik}+N_{jk}}\left[N_{ik}z_{ik}+N_{jk}z_{jk}\right],k=1,2,\ldots ,L$$

In Equation 6, the two merged cluster center vectors are, respectively, weighted by the number of samples in their clustering domain, so that $z_{k}^{*}$ is the average vector of the true proof.

##### 2.4.1.6 Step 6: iteration termination

If it is the first iteration, the algorithm ends; otherwise, change the input parameters and go to (1) of the first step; if the input parameters do not change, go to (2) of the first step, and the number of iteration operations should be increased by each time.

#### 2.4.2 Evaluating clustering effects

To consider the superiority between the two clustering effects and the desirability of k -values, the Elbow Rule and Silhouette Coefficient are used to evaluate them. The Elbow Rule’s core idea is that the larger the categorical number k is, the finer the sample division will be, the aggregation degree in each category will gradually increase, and sum square error (SSE) will gradually become smaller. By calculating the sum square error under different cluster number k to obtain the cluster number comparison graph, observe the trend of the graph, when the value of k decreases, the corresponding SSE value will also decrease gradually, when the value of k increases gradually, the SSE value also decreases gradually until it tends to be stable, and the whole SSE change curve presents a shape similar to an elbow. To determine the optimal cluster number, it is necessary to find a k -value in the graph, when the k -value increases with a larger decline in SSE, and after the k -value increases the trend of the curve decreases gradually flatten out to find the inflection point in the whole curve, then the corresponding k -value is the optimal number of clusters (39).

For a sample set, its Silhouette Coefficient is the average value of all sample Silhouette Coefficients, which takes the value range of [−1,1], the closer the distance of the same feature sample cluster, the further the distance of different sample clusters, the higher the Silhouette Coefficient, the closer the value is to 1, which indicates that the clustering effect is better, on the contrary, the closer the value is to −1, which indicates that the clustering effect is poorer. When the Silhouette Coefficient is 0, it indicates that there are overlapping clusters. Therefore, calculating the sample set Silhouette Coefficient can reflect the clustering effect more accurately.

The Silhouette Coefficient *S* for a single sample is *S_i_*, and the calculation formula for *S_i_* is Equation 7:


(7) $$S_{i}=\frac{b_{i}-a_{i}}{\max\left(a_{i},b_{i}\right)}$$

In Equation 7, where a is the average distance of a given sample from all other samples within the same cluster, and b is the average distance of a given sample from all samples in the nearest neighboring cluster (i.e., the smallest average distance to samples in another cluster).

The average Silhouette Coefficient *SC* of the sample set is calculated according to Equation 8 as follows:


(8) $$SC=\frac{1}{N}\sum_{i=1}^{N}S_{i}$$

where N is the total number of samples, and $S_{i}$ is the Silhouette Coefficient for the i -th sample.

#### 2.4.3 Analyzing the organization coefficient for human stampede of mass gathering

In order to explore the influence of the organizational pattern on the risk factors of human stampede in mass gathering activities, the organization coefficient is proposed to characterize the type of organization of the activities in different clusters, using the percentage on the samples in different organizational patterns per cluster and the percentage on the whole dataset as a ratio to indicate the organization type in each cluster for the sample activities, and the size of the samples with different organizational patterns per cluster can be used to indicate the organizational patterns of the activities within that cluster.


(9) $$O_{ki}=\frac{p_{ki}}{P_{i}}$$

where $O_{ki}$ is the organization coefficient of cluster k under the organizational pattern i . i is the organizational patterns number, i =1 ~ 3 refers to organized type, applied type and spontaneous type. The higher the $O_{ki}$ , the more obvious the characteristics of the organizational pattern i are exhibited in cluster k . Based on this, the organization characteristics of the cluster can be judged. $p_{ki}$ is the proportion of samples with different organizational patterns in each cluster. $P_{i}$ is the proportion of samples with different organizational patterns in the dataset.

## 3 Result

### 3.1 Cluster partitioning and clustering effect evaluation

ISODATA algorithm is used for clustering analysis, and MATLAB software is used to process the 209 * 15 eigenvector set. The selection range of clustering centers is k =2 ~ 10. The results show that when k =8, the contour coefficient of each cluster exceeds 0. However, 6 cases of “no cluster structure” were found, which indicates that the clustering effect is not ideal when using 15 variables, and the number of variables needs to be reduced (62). Therefore, Balbi (63) p proposed a method to eliminate irrelevant variables to reduce the dimension of the database. Consequently, in this study, certain variables pertaining to the human stampede accident context (time period, place layout characteristics, place openness characteristics, and crowd structure characteristics) were excluded. Subsequently, the cluster analysis was rerun, resulting in a notable enhancement of the calculated Silhouette Coefficient for clustering. The data are shown in Table 4, where the maximum Silhouette Coefficient is 0.4235, and the maximum average Silhouette Coefficient is 0.3018.

**Table 4:** Cluster Silhouette Coefficients after ISODATA algorithm dimensionality reduction.

Clustering center *k*	Clustering Silhouette Coefficient	Average silhouette value of each cluster	“No cluster structures” numbers (average silhouette value <0.25)
2	0.1736	0.1836;0.1617	
3	0.1969	0.1965;0.1462;0.2704	2
4	0.2125	0.1925;0.1586;0.2704;0.2449	3
5	0.2223	0.1932;0.1730;0.31010.2560;0.2781	2
6	0.2535	0.2276;0.2042;0.4088;0.2768;0.2186;0.2172	4
7	0.2583	0.1816;0.2071;0.3476;0.3367;0.3256;0.2286;0.2546	3
8	NaN	NaN	/
9	0.2913	0.2384;0.2962;0.4551;0.3389;0.3408;0.2768;0.2940;0.2551;0.2244	2
10	0.3018	0.2811;0.2875;0.3410;0.3477;0.4235;0.2768;0.2948;0.2748;0.2475;0.2722	1
11	NaN	NaN	/

**Figure 1:**
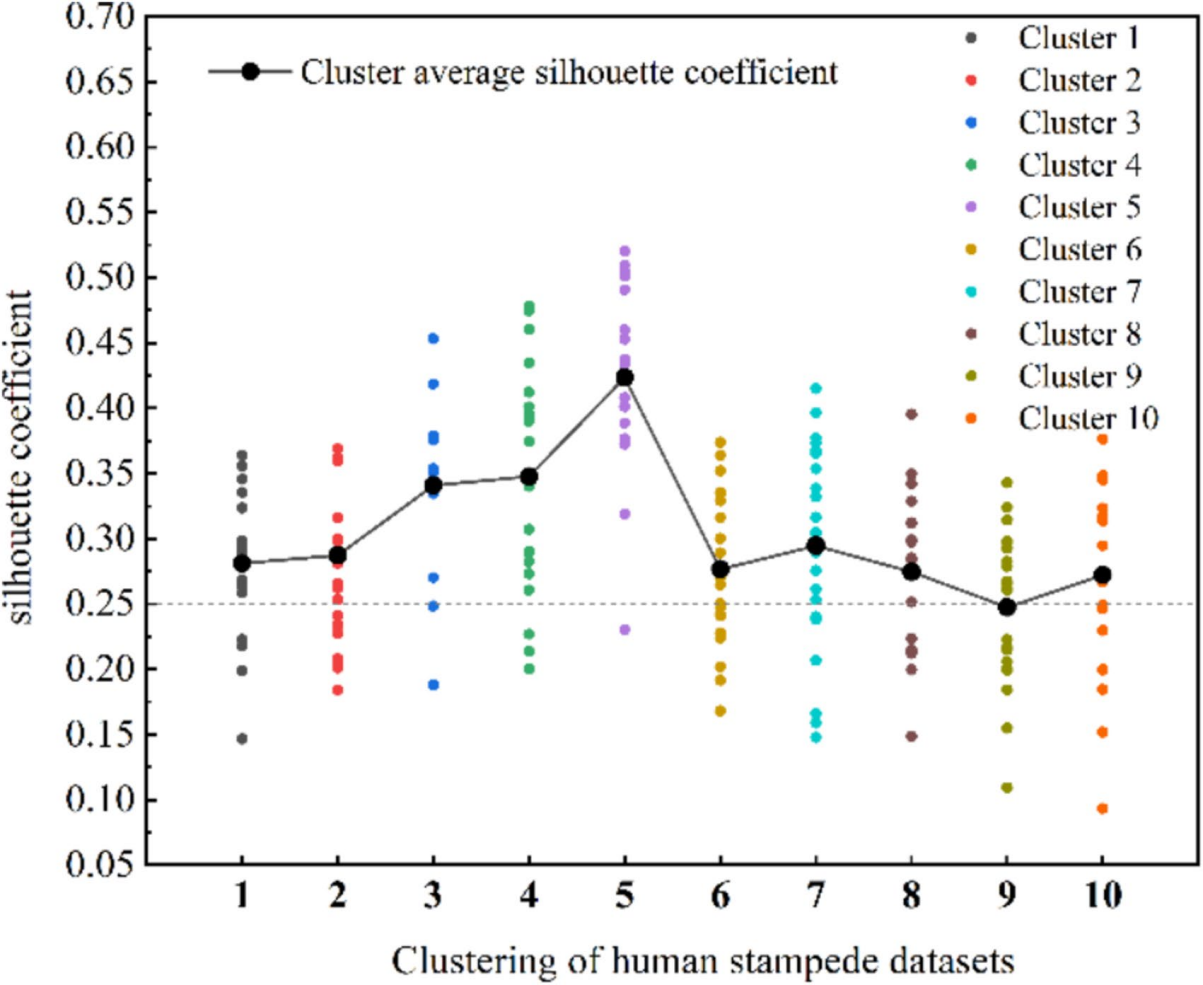
Distribution of Silhouette Coefficients after dimensionality reduction (*k* = 10).

The optimal result was obtained from ISODATA after downscaling, with a cluster center at k =10 following dimensionality reduction. Detailed results of the optimal clusters are depicted in Figure 1. This outcome showcases a superior clustering structure characterized by Silhouette Coefficients surpassing 0 within each cluster. Additionally, the significant decrease in cluster-free structures underscores a well-organized clustering arrangement, affirming the effective classification of the dataset.

### 3.2 Identification of three organizational pattern clusters

**Figure 2:**
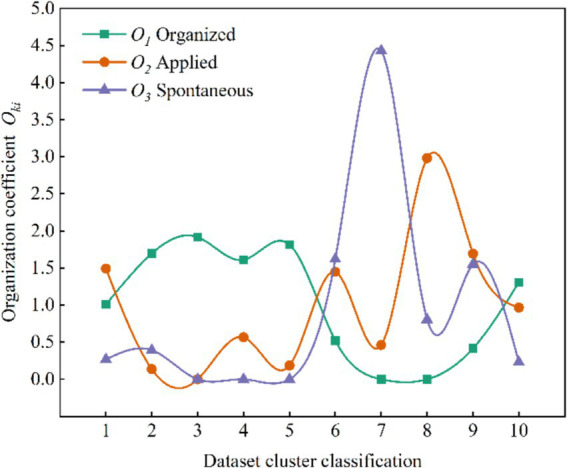
Distribution of results of organization coefficient calculations.

Calculate the percentage of different activities organizational pattern in each cluster and the whole dataset for the sample number, according to the Equation 9, the distribution of organization coefficient $O_{ki}$ in 10 clusters is obtained. $O_{ki}$ is the organization coefficient of cluster *k* under the organizational pattern i . Table 5 and Figure 2 shows the results and distribution of $O_{ki}$ respectively.

**Table 5:** Summary of organization coefficient results.

Cluster No.		Organization coefficient(*O_ki_*) of each cluster under different organizational patterns		
		*O*_*k*1_	*O*_*k*2_	*O*_*k*3_
Cluster 1	*O*_1*i*_	1.00917	1.49153	0.26829
Cluster 2	*O*_2*i*_	1.69619	0.13625	0.39212
Cluster 3	*O*_3*i*_	1.91743	0	0
Cluster 4	*O*_4*i*_	1.61064	0.56678	0
Cluster 5	*O*_5*i*_	1.81651	0.18644	0
Cluster 6	*O*_6*i*_	0.52294	1.44915	1.62195
Cluster 7	*O*_7*i*_	0	0.46205	4.43266
Cluster 8	*O*_8*i*_	0	2.98305	0.80488
Cluster 9	*O*_9*i*_	0.41683	1.69418	1.55143
Cluster 10	*O*_10*i*_	1.30734	0.9661	0.23171

According to Figure 2 and Table 5, Cluster 7 is identified as a prototypical consolidation of spontaneous activities that contribute to human stampede in mass gathering activities. This attribution is reinforced by its possession of the highest organization coefficient ( $O_{73}$ _=_4.4327) when the organizational pattern is the spontaneous type ( i =3). Cluster 8 is recognized as a representative assembly characterized by the applied type, displaying a relatively higher organization coefficient ( $O_{82}$ =2.9831) under the applied organizational pattern ( i =2). Cluster 3 is identified as organized types. This is evident in their possession of the highest organization coefficients under i =1, with $O_{31}$ =1.91743. Upon consolidating the results, Clusters 3 as organized clusters, Cluster 8 as applied cluster, and Cluster 7 as spontaneous cluster.

### 3.3 Cluster characteristics for each organizational pattern

#### 3.3.1 Risk characterization of human stampede in the organized activities

**Figure 3:**
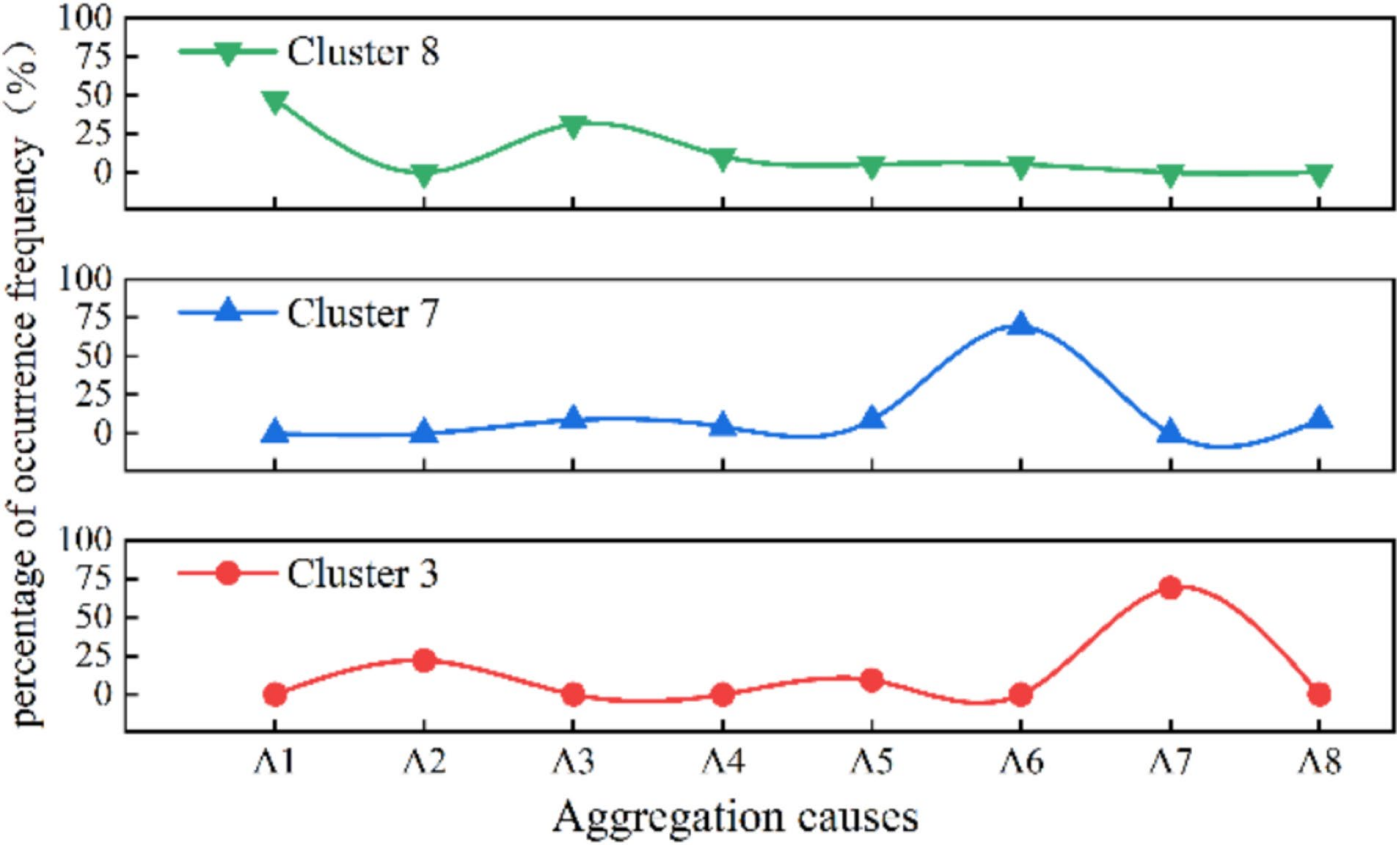
Distribution of the aggregation causes in the three organizational patterns of clusters (%).

Figure 3 illustrates the distribution of the characterization contributing to the risk factors under aggregation causes in organized type of human stampede accidents. The primary cause for crowd gathering in Cluster 3 is “A7 education and training,” constituting 69.21% of events in the cluster. Which is significantly higher than the overall dataset average (Difference > 10%). During the mass gathering activities, the number of tourists motivated by parent–child research, family leisure and cultural experience increased significantly, which prompted more education and training activities to be combined with tourism, forming organized learning exchange and training activity. This phenomenon is particularly obvious in China (64). Similarly, the proportion of “A2 sports events” is 21.89%, which is basically consistent with the results of single factor analysis, indicating that some sports events have organizational characteristics.

**Figure 4:**
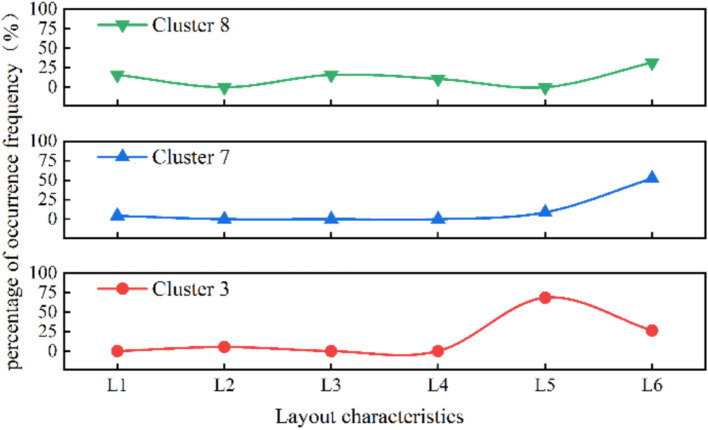
Distribution of layout characteristic in the three organizational patterns of clusters (%).

Figure 4 shows the risk factors under layout characteristic in organized type of human stampede. It can be seen that in Cluster 3, the predominant layout characteristic of human stampede is concentrated in “L5 stairs” (68.42%), significantly surpassing the corresponding proportion in the overall dataset (17%). Additionally, there is a notable high proportion of the factor “L6 entrance and exit” (26.32%), and the layout characteristics in single factor analysis are mainly entrance and exit, which are consistent.

**Figure 5:**
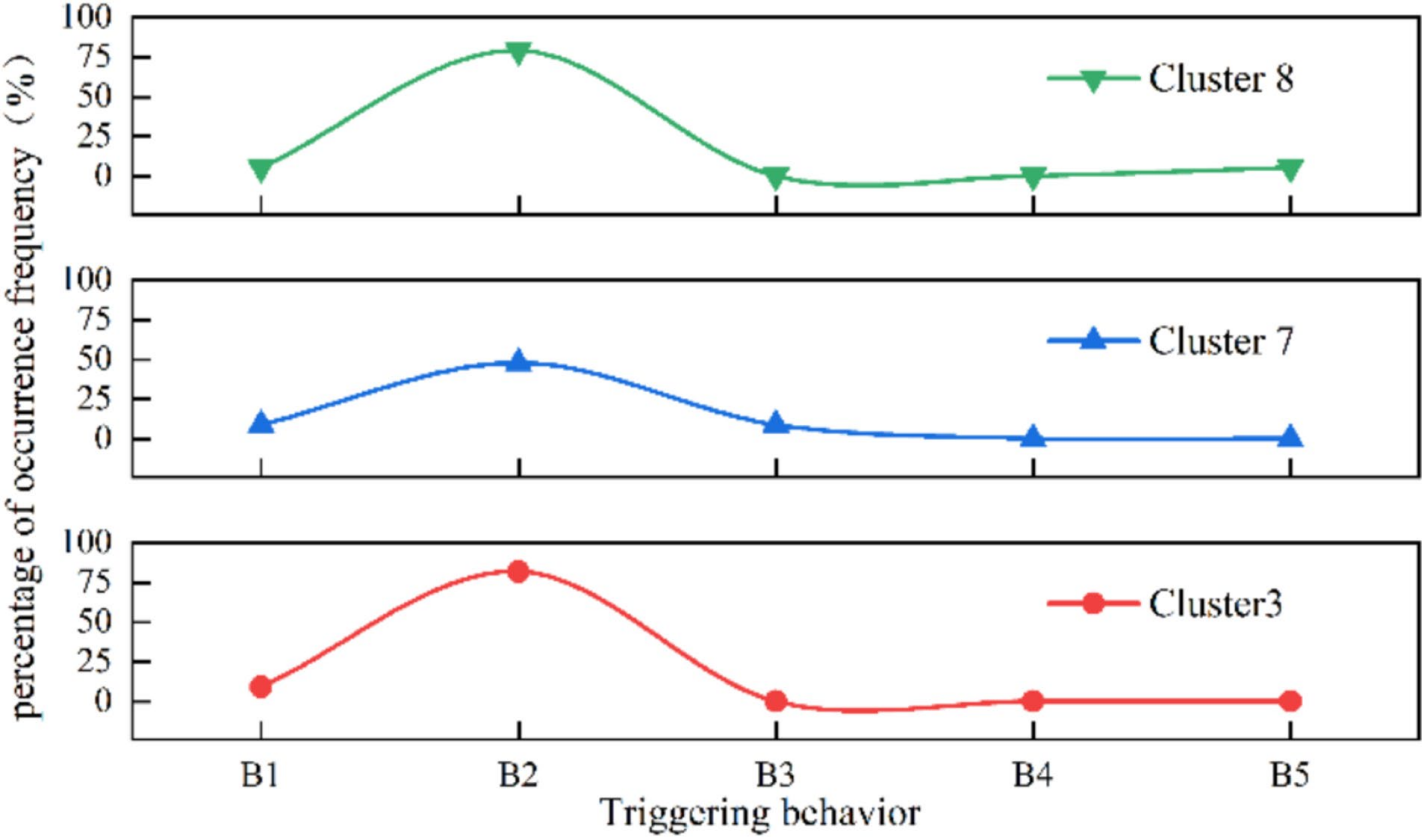
Distribution of triggering behavior in the three organizational patterns of clusters(%).

**Figure 6:**
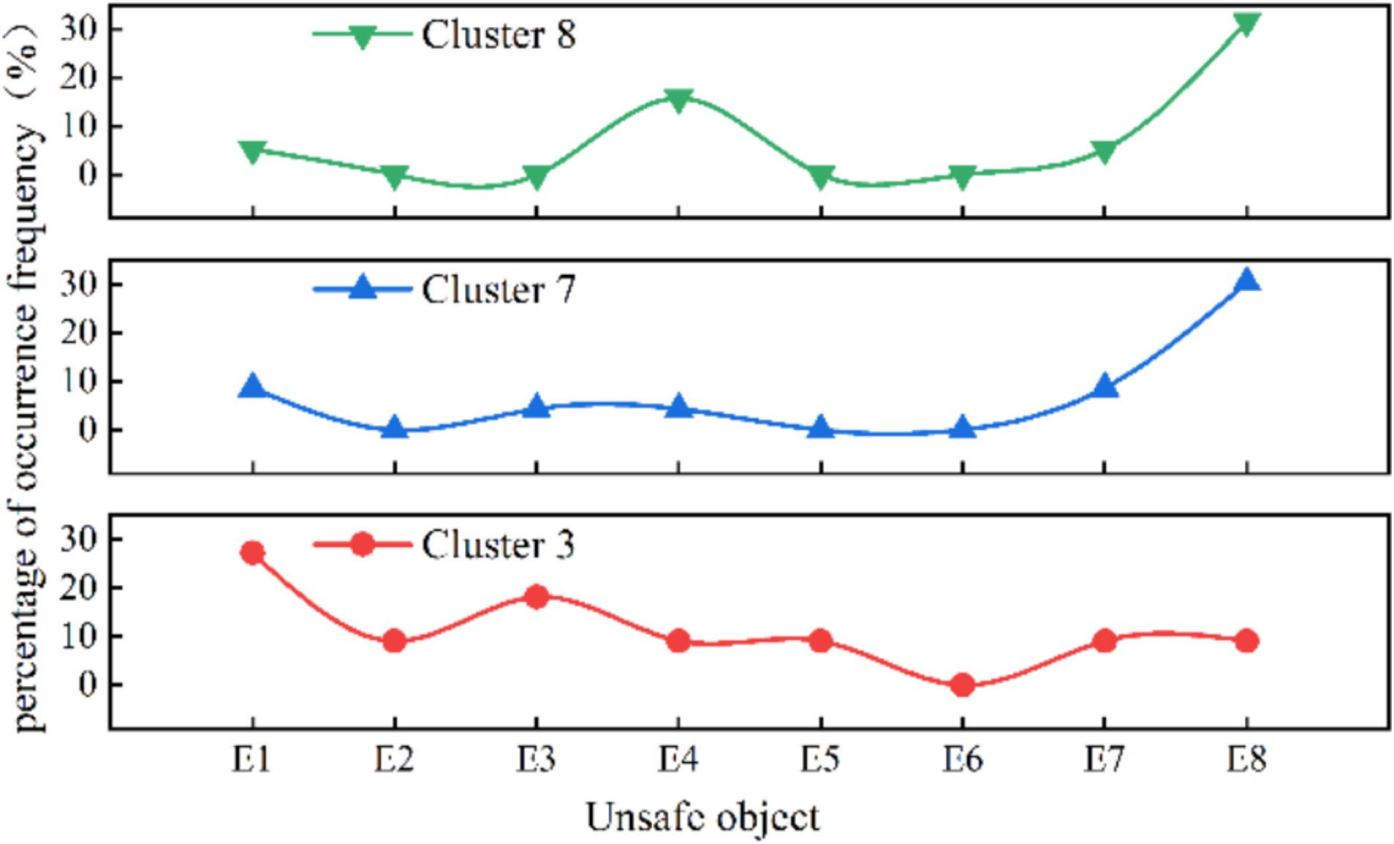
Distribution of unsafe object in the three organizational patterns of clusters (%).

In the analysis of other risk characteristics for cluster 3 (Figures 5, 6), at the risk factors of direct cause level, the highest proportion of unsafe behaviors in cluster 3 is “crouching, falling” (68.42%), and the highest proportion of unsafe objects are “unreasonable number and width of safety exits” (27.27%) and” sudden disaster accidents “(26.32%) respectively. At the risk factors of indirect causes level, the three aspects of knowledge, awareness, and psychology in Cluster 3 accounted for the highest proportion of “weak emergency response skills in accidents” (36.36%), “poor public safety concepts” (72.72%), and “panic mentality” (9.09%), respectively. At the risk factors of root cause level, “poor crowd dispersal at the scene” constitutes 10% in Cluster 3.

#### 3.3.2 Risk characterization of human stampede in the applied activities

As the applied type of human stampede, Cluster 8 has the primary aggregation causes of “religious activities” (47.37%) and “celebrations” (31.58%), constituting 12 and 16% of the dataset, respectively. In terms of layout characteristics, the predominant factor in Cluster 8 is “entrances and exits” (31.58%), followed by “narrow places” and “alleys,” each accounting for 15.79%.

According to Figures 5, 6, the analysis of other risk characteristics reveals that in Cluster 8, the primary triggering behavior is “falling, squatting” (78.95%), and the most significant unsafe object is “overloading of premises capacity” (15.79%) among the direct cause risk factors. In the analysis at risk factors of indirect causes, the leading knowledge factor is “insufficient awareness of accident hazards” (68.42%), while the dominant awareness factor is “weak public safety awareness” (78.95%). Regarding psychological factors, the factor with the highest proportion is “competitive psychology” (21.05%). At the risk factors of root cause level, the root cause indicates that “weak on-site crowd management” (31.58%) holds the highest share in Cluster 8, which is significantly higher than the overall dataset average (Difference > 10%).

#### 3.3.3 Risk characterization of human stampede in the spontaneous activities

As the spontaneous type of human stampede, the highest proportion of aggregation causes in cluster 7 is “charity promotions” (69.57%), followed by “concerts” and “festivals.” The layout of venue where human stampede occurred is mainly characterized by “entrances and exits” (52.17%). At the risk factors of direct cause level, the triggering behaviors is mainly manifested by “falling and crouching” (47.82%), and the unsafe physical state was manifested by “overload in the place” (30.43%).

At the level of indirect cause risk factors, in terms of knowledge factors, the ratios of “lack of awareness of accident hazards “(47.83%) and “weak accident response skills” (43.48%) are similar and both are higher than the percentage of single factors. In terms of awareness factors, the order is “weak public safety awareness” (69.57%), followed by “poor emergency response” (26.09%). In terms of psychological factors, the proportions of “competitive mentality” (65.22%) and “panic mentality” (21.74%) are relatively high. At the level of root cause risk factors, the factors contributing to the significant proportion were “weak crowd management capacity” (34.78%) and “poor crowd dispersal at the scene” (26.09%).

## 4 Discussion

### 4.1 Divergences analysis of mass gathering under different organizational patterns

**Figure 7:**
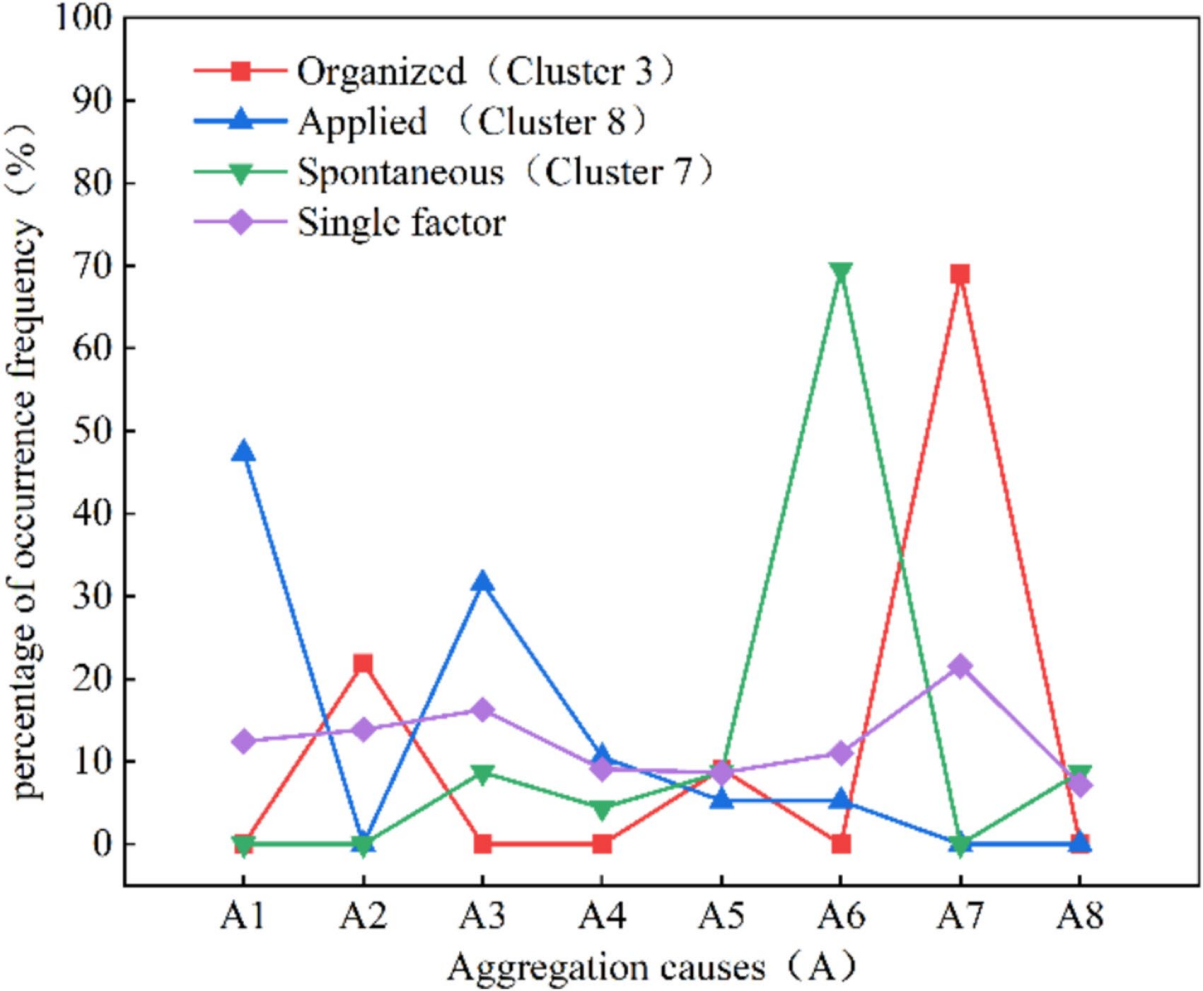
Aggregation causes percentage distribution curve comparison.

Among the 15 major categories of risk factors in Table 2, three types of risk factors under aggregation causes, layout characteristics and risk levels exhibit significant differences in various organization situations. Figure 7 illustrates a comparison of the aggregation causes across the three organizational patterns. The proportion of sports events(A2) education and training events (A7) occurring is high in organized clusters, primarily due to many sports events are arranged during holidays to encourage and attract people to participate in large-scale sports events. In addition, the promotion of sports events is often regarded as part of the tourism package, because they can enhance the overall tourism experience. The aggregation causes in applied type predominantly involve religious activities (A1) and celebratory events (A3). In spontaneous type, the prevalence of charity and promotional events (A6) significantly outweighs that of single-dimensional activities.

The findings indicate that although there is minimal disparity in aggregation causes within the overall sample. However, significant distinctions become apparent when examining different organizational patterns of mass gathering. This variation provides compelling evidence for tailoring preventive measures to address specific types of activities. Although scholars have generally overlooked variations in the causes of aggregation associated with different organizational patterns, certain researchers focus on the analysis of sport events overcrowding cases (8, 13), which is a typical organized type. Conversely, others concentrate on religious activities (20), mostly characterized as an applied type. Furthermore, there are scholars studying specific accidents such as the “Love Parade” in Germany (45, 58, 59), “10.29” Itaewon stampede accident (6, 65), and the “12.31” stampede in Shanghai Bund (15, 66), etc., which are typical examples of spontaneous human stampede accidents. This suggests gatherings motivated by diverse reasons may exhibit distinct risk characteristics in human stampede accidents. The research presented in this article systematically and quantitatively elucidates this distinction, underscoring its empirical validity.

**Figure 8:**
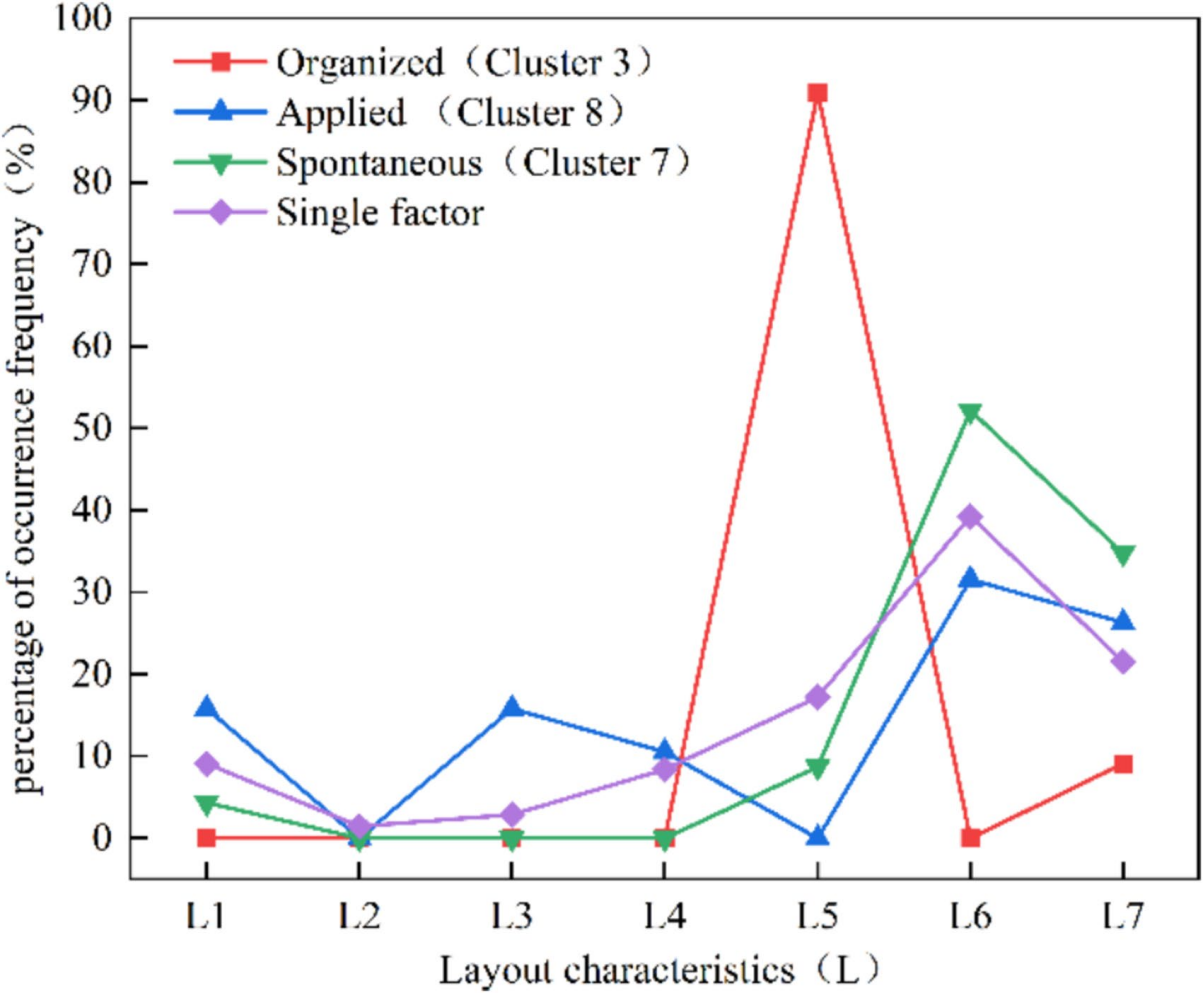
Layout characteristics percentage distribution curve comparison.

Figure 8 illustrates a comparison of risk factors under layout among the three organizational patterns. It reveals similarities in layout features between the applied and spontaneous types of human stampede in mass gathering activities, yet significant distinctions emerge when compared to the organized type. Human stampede of organized activities typically takes place on stairs (L5). The research findings of pertinent scholars substantiate this assertion (14). Zhang and Zhou (13) observed that stairs and platforms are the most common locations for human stampede accidents, accounting for 92.3% of cases. This congestion is mainly caused by the narrow corners of stairs, which increase the risk of tripping or falling. These incidents often force people to stop or slow down abruptly, disrupting movement and leading to human stampede.

The applied and spontaneous types primarily occur at entrances and exits (L6). Illiyas et al. (20) in their analysis of religious activities, identified potential safety hazards in the design of event venues. During the conclusion of events or the onset of surges at the beginning, crowds tend to exit or enter, and the bottlenecked exits intensify congestion, ultimately contributing to accident.

In spontaneous activities, the absence of dedicated organizers and a potential lack of staff near entrances and exits can result in inadequate guidance and increase the risk of human stampede. Helbing et al. (23) identified one of the factors contributing to the accident at the Duisburg Love Parade as tourists using narrow stairs as potential emergency entrances and exits. When tourists attempted to reach these areas, the sudden increase in crowd density triggered a human stampede accident. Moreover, fluctuations in crowd psychology and emotions are particularly concentrated during the entry into and exit from activity areas, inevitably giving rise to anxious or impatient emotions, thereby increasing the likelihood of conflicts.

**Figure 9:**
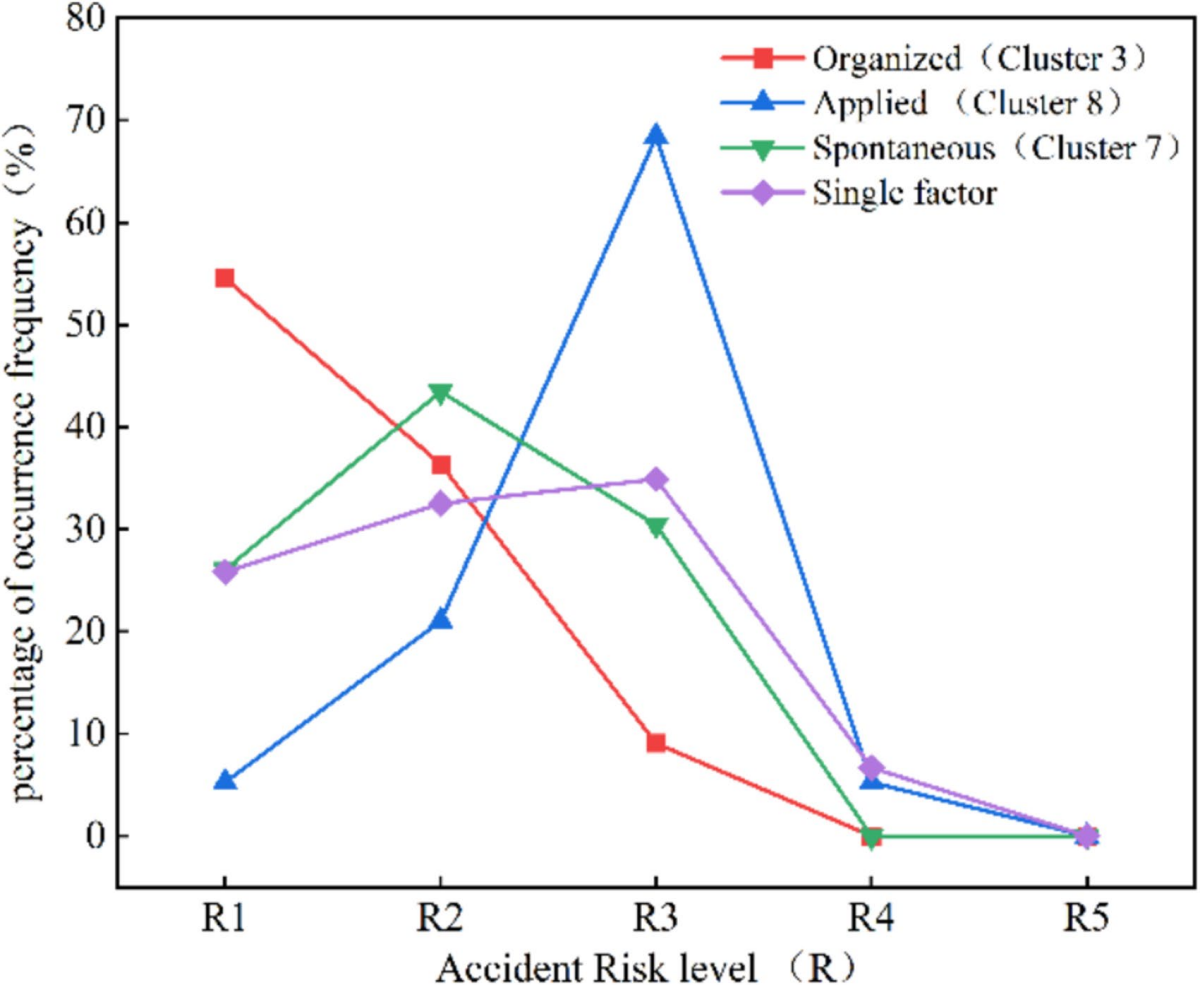
Accident risk level percentage distribution curve comparison.

Figure 9 presents a comparative analysis of human stampede accident consequences across various organizational patterns. The results reveal that the risk levels of the applied type are largely concentrated in R3 (from 10 to 100 people died), indicating that this type of gathering activity is most prone to result in extensive casualties and injuries. The spontaneous type primarily concentrates in R2 (from 0 to 10 people died), while the organized type exhibits distributions in both R1 (none died)and R2.

The elevated risk level associated with the applied type can be attributed to the religious activities of this cluster. The substantial participation in religious events leads to heightened crowd density, increasing the potential for human stampede. Ahmed and Memish (19) notes that, according to a report from the Pew Research Center on the Global Religious Landscape, over 5.8 billion individuals worldwide, including both adults and children, are affiliated with religious beliefs. As event venue expands, security and rescue capabilities in remote areas tend to be relatively inadequate, and rescue and evacuation efforts become significantly challenging (20). Religious crowds often experience drastic emotional shifts, making it easier to lose control and succumb to frenzied or panicked conditions (67). At times, organizational and management capabilities struggle to keep pace with the rapid influx of participants, resulting in insufficient responses and chaotic emergency handling. Furthermore, certain religious doctrines emphasize sacrificing for faith, diminishing the likelihood of individuals taking proactive measures to escape danger. Hence, the intricate psychology of crowds within religious gatherings is a point that requires further research and attention in mass gathering activities involving human stampedes.

The risk level in spontaneous activities is relatively lower compared to the applied type, primarily owing to the diverse demographic composition of participants and the absence of strong shared beliefs and emotional bonds among them. However, group psychological effects within the population can still induce irrational behavior under pressure. Spontaneously assembled crowds demonstrate greater autonomy and randomness, moving according to their own preferences. This randomness implies that participants may struggle to predict the behavior of others, and such unpredictability can potentially trigger panic or dangerous behavior in emergency situations. Additionally, the venues for such activities are typically public spaces, characterized by openness and dispersion, as opposed to the closed and densely areas (24). These spaces may not have taken into account the human stampede in mass gathering activities during the design phase owing to their early construction period.

While various measures can be suggested to enhance the safety management of spontaneous activities, such as improving the safety design of public spaces, disseminating emergency information, and reinforcing public safety education. However, there is a need for more in-depth research on the characteristics and evolutionary mechanism of risk factors in spontaneous activities. This includes exploring the complex factors and coupling mechanisms of spontaneous crowd aggregation, examining the influence of crowd psychology on spontaneous behavior. Furthermore, even in spontaneous activities should encourage clear safety responsibility principles, which is conducive to contributing to the enhancement of safety management and risk prevention measures for sustainable travel.

The risk levels of the organized activities primarily concentrated around R1 and R2. Such activities adhere to explicit organizational disciplines, rules, and regulations, indicating that participants exhibit a high degree of self-discipline in following the commands and instructions provided by the organizers (14). Consequently, the likelihood of extreme emotional responses or destructive behaviors is mitigated. Hence, from an organizational perspective, the risk prevention and control system for such activities appears notably comprehensive. However, unsafe behavior may still occur in specific situations, such as sudden events causing panic or chaos, where participants may instinctively take self-protection actions that could violate established safety rules. Additionally, unclear or untimely communication of instructions and rules may lead to individual participants misunderstanding or not receiving information, resulting in unsafe behavior. Therefore, emergency drills are strongly recommended for spontaneous activities.

### 4.2 Similarities analysis of mass gathering under different organizational patterns

**Figure 10:**
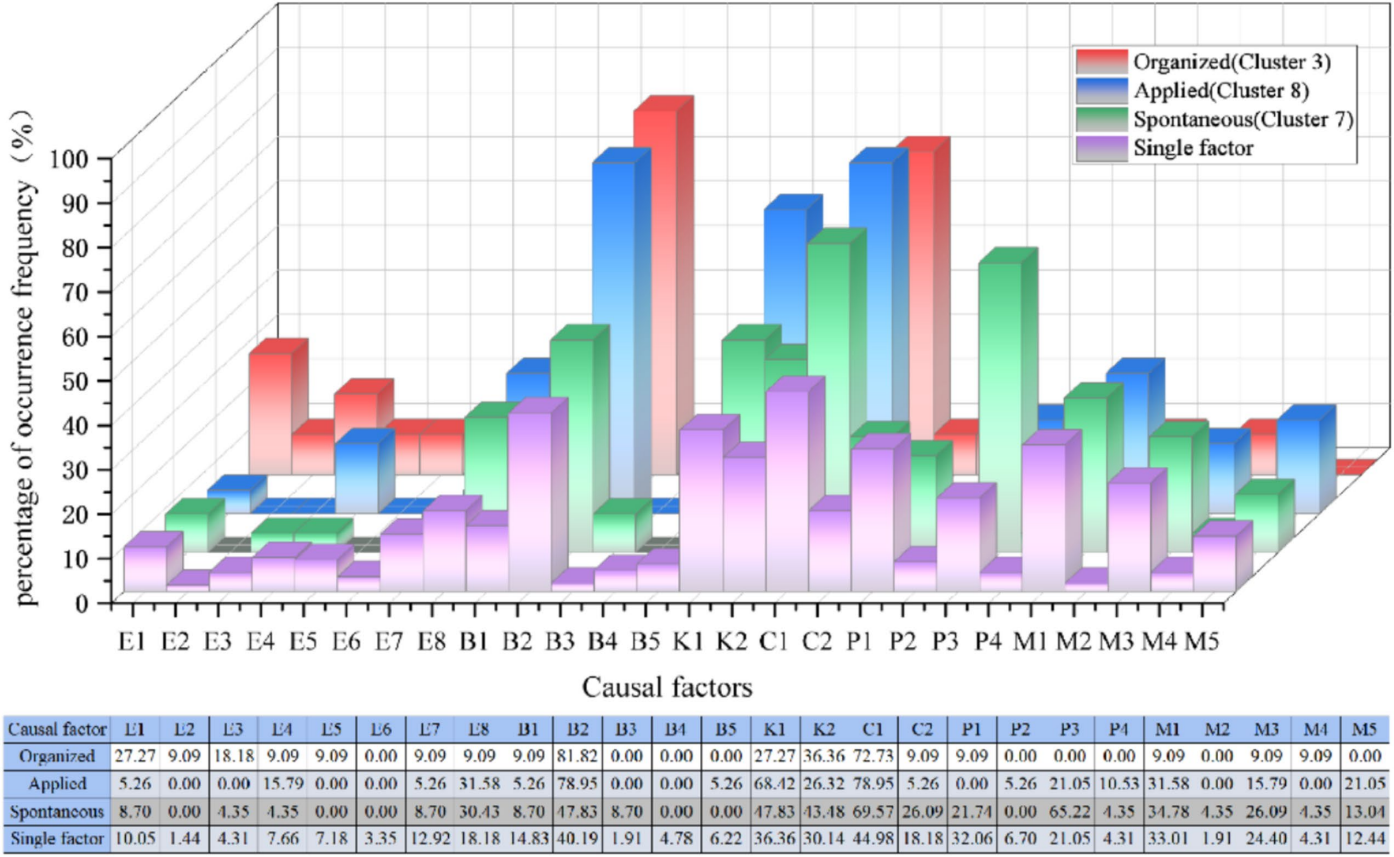
Other six accident cause factors percentage distribution curve comparison.

Figure 10 depicts the distribution of the other six major categories of risk factors contributing to the risk characterization of human stampede in mass gathering activities under the three organizational patterns. The factor codes corresponding to these categories are provided in Table 2.

It is observed that the risk factors of the four categories, namely trigger behavior (B), knowledge (K), consciousness (C) and management (M), exhibit similarities in the distribution of risk characteristics across different organizational patterns. Within the triggering behavior category (B), factors B1 (quarrel, fight and terrorist attack) and B2 (fall) constitute a significant percentage in all clusters. Knowledge factor K2 (weak accident response skills) and consciousness factor C2 (poor emergency preparedness), along with management factors M1 (weak crowd management ability), M3 (poor on-site crowd evaluation), and M5 (insufficient safety measures), demonstrate a relatively high frequency distribution. This indicates that, although the organizational patterns in mass gathering activities may vary, the challenges related to personal characteristics and crowd management are analogous.

We have analyzed the reasons for this phenomenon and posit that conflicts and fights between individuals can arise due to interpersonal conflicts, misunderstandings, or tensions, irrespective of the organizational patterns of activities (68). Such occurrence is notably prevalent in crowded places, where individuals are at an increased risk of getting involved in conflicts, leading to the presence of B1 in all clusters. Additionally, factors such as slippery and uneven terrain can elevate the likelihood of falls (B2), a risk that is not confined to any specific organizational patterns.

The shared existence of factors K2 and C2 in knowledge and awareness underscores the critical role of education and training in individuals’ ability to handle emergencies. Issues may arise in any cluster scenario if individuals lack training in emergency response skills, awareness of emergency preparedness, and necessary self-rescue skills in their daily environment (69). Therefore, prioritizing comprehensive safety training and emergency preparedness education for individuals involved in various activities can effectively prevent overcrowding from turning into stampede accidents.

### 4.3 Strategies for mass gathering under different organizational patterns

**Figure 11:**
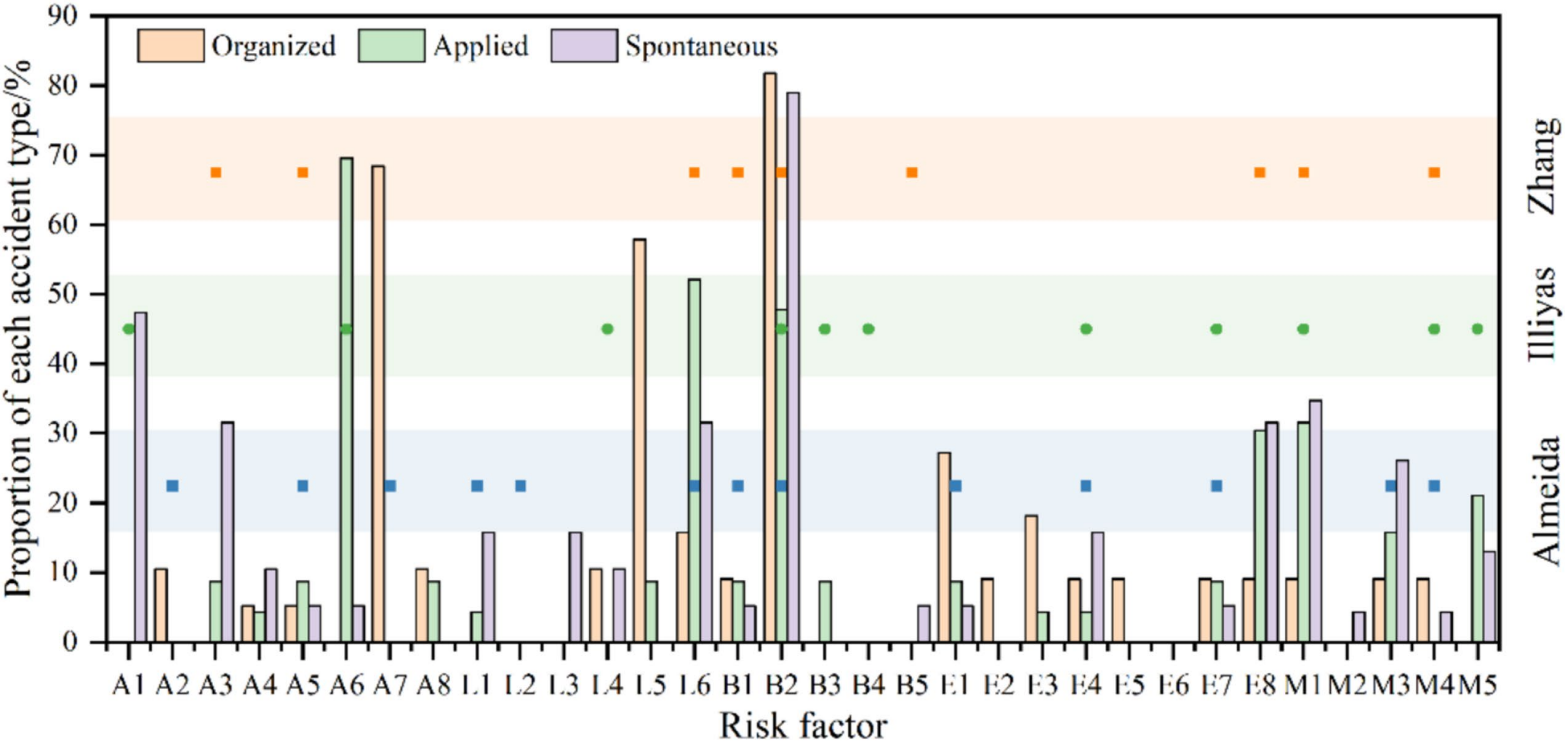
Comparison of risk factor proportions across different studies.

Based on an analysis of the similarities and differences in risk characteristics across different organizational patterns, we developed Figure 11 to illustrate the theoretical foundation for formulating safety strategies for the three types of activities. Figure 11 presents the proportion of risk factors across the three type activities and compares them with those identified in prior studies on crowd gatherings. While previous studies on risk factors in crowd gatherings provide a valuable foundation for developing safety strategies, their selection of risk factors is often activity-specific and fails to account for the influence of organizational patterns. For instance, Almeida et al. (59) focused on the impact of venue design and crowd density in specific religious gatherings, while Illiyas et al. (20) emphasized the role of rumors and temporary structure collapses in religious festival stampedes.

Compared to other studies, our research offers a comprehensive analysis of risk factors across three organizational patterns: organized, applied, and spontaneous activities (Figure 11). For instance, for layout character (L), organized activities predominantly occur on “stairs” (L5; 68.42%), while applied and spontaneous activities primarily occur at “entrances and exits”(L6; 31.58, 52.17%). For triggering behaviors (B), “falling and squatting” (B2) is prominent across all activity types: organized (68.42%), applied (78.95%), and spontaneous (47.82%). For unsafe objects (E), organized activities are characterized by “unreasonable number and width of safety exits” (E1; 27.27%) and “sudden disaster accidents” (E7; 26.32%). Applied activities show “overloading of premises capacity” (E8; 15.79%), while spontaneous activities highlight “overload in the place” (E8; 30.43%), emphasizing overcrowding risks. These findings underscore the varying significance of risk factors across organizational patterns, offering a more nuanced and systematic perspective than the activity-specific focus of earlier research. By integrating Grounded Theory and ISODATA, our study delivers novel insights into risk characteristics across organizational patterns, establishing a foundation for further exploration of safety strategies.

For organized activities, characterized by high-density crowds and specific spatial layouts (e.g., staircases, narrow passages), we recommend employing crowd flow models and simulations to predict and manage density. Venues should incorporate wide staircases, sufficient emergency exits, and clear signage to ensure safe movement. Regular drills and training for staff and participants are also essential for effective emergency response. These recommendations build on the strategies of Ahmed and Memish (19) and Koski et al. (29), offering tailored solutions for organized activities.

For applied activities, such as religious gatherings and celebrations, dynamic crowd management strategies are critical. Real-time monitoring systems (e.g., video surveillance, drones) are recommended to track crowd density and movement. As for spatial layout, our findings reveal that organized activities face higher risks on staircases, while applied and spontaneous activities are more vulnerable at entrances and exits. Therefore, we propose optimizing safety exit layouts to enhance evacuation routes, implementing staggered entry and exit times to reduce congestion, and ensuring security personnel and volunteers are trained in crowd management and emergency response.

For spontaneous activities, defined by their lack of formal organization and inherent unpredictability, flexible emergency response plans and targeted public awareness campaigns are vital. Public awareness initiatives should focus on educating participants about crowd safety protocols and emergency procedures. Emergency response plans must be adaptable to rapidly changing conditions, while designated safe zones should be established to provide refuge during emergencies. These recommendations are targeted at the unique risks associated with spontaneous gatherings, contrasting with prior studies that predominantly focus on planned events.

It is noteworthy that mutual conversion between activity types may occur. Traditional Eastern celebrations, such as religious festivals, temple fairs, and cultural events, often exhibit characteristics of both applied and spontaneous activities. While these activities are typically organized by religious or community groups (aligning with the applied type), they may also attract spontaneous participants who join without formal organization. This dual nature poses unique challenges for safety management, necessitating comprehensive strategies for crowd control, emergency response, and educational training. These findings highlight the importance of developing adaptive safety frameworks that account for the dynamic organizational patterns of mass gatherings. Future research could explore the effectiveness of specific interventions, such as real-time crowd monitoring technologies or targeted public awareness campaigns, in mitigating risks associated with hybrid activity types.

## 5 Conclusion

This study proposes a method combining Grounded Theory and Unsupervised Clustering Algorithms to explore the impact of organizational pattern on human stampede of mass gathering. In order to break the traditional framework of accident causation analysis and solve the problem that traditional causation analysis methods are not detailed, Grounded Theory was used to code the text, and a comprehensive and detailed accident causal set was constructed with 3,186 sentence descriptions, 15 core categories, and 57 sub-factors extracted from 209 human stampede accident texts. With the purpose to further explore the characteristics of risk factors of human stampede in mass gathering activities under different organizational patterns, the organization coefficient is proposed to classify the organizational patterns in clustering results to determine three types of clusters including organized, applied, and spontaneous, which facilitates to carry out the comparative analysis of the differences in risk factors and safety management across various mass gathering activities.

It is found that there are differences in three organizational patterns of clusters in terms of aggregation reasons, layout and risk level through the comparative analysis of human stampede accidents under different organizational patterns. The findings indicate that while there is minimal variation in aggregation causes across the overall sample, these variations becomes significant under different organizational patterns, particularly in education and training activities, religious events, and celebrations Additionally, the research identified noticeable layout differences in activities of different organizational patterns, such as organized clusters occurring more frequently on staircases, while applied and spontaneous clusters mainly took place at entrance and exit. In terms of risk levels, applied type clusters had a higher risk of accidents, resulting in relatively more casualties. Furthermore, the study recognized commonalities present across different organizational patterns, such as triggering behaviors, knowledge, awareness, and management. This research, by exploring the heterogeneous of organization patterns in mass gathering activities, provides valuable theoretical support for enhancing safety management and risk prevention strategies in mass gathering activities. The results emphasize the importance of tailored safety measures based on the specific characteristics of organizational patterns, ultimately supporting the effective management of crowd safety in mass gathering activities.

## Data Availability

The original contributions presented in the study are included in the article/supplementary material, further inquiries can be directed to the corresponding authors.
